# Supplementary material for: Unveiling the spectrum of Arabic offensive language: Taxonomy and insights
Source: PLoS One. 2025 Jun 17;20(6):e0319900. doi: 10.1371/journal.pone.0319900 (PMC12173391; doi:10.1371/journal.pone.0319900)
Supplement: S1 Appendix — (PDF) [file pone.0319900.s001.pdf]

S1 Appendix

We present an organized description of related work on detecting offensive language in Arabic. A detailed discussion of these works is provided in Section 2.1. S1 Table 1 summarizes the papers we review, including their publication year, methods, features, datasets, and key outcomes.

S1 Table 1. Summary of Arabic offensive language detection studies

ref & year	methods	features	dataset	results
[2] 2020	Bi-GRU, GRU, LSTM, RNN	Offensive text features in social media	SemEval 2020 shared task dataset [17]	Best performance achieved with SalamNET
[4] 2020	Ensemble machine learning	linguistic and statistical features	OSACT4 [44]	Ensemble method outperformed single learners
[5] 2020	CNN, Bi-GRU with attention layer	Deep neural features	SemEval 2020 dataset [17]	Best results achieved with Bidirectional GRU
[6] 2022	BERT, SVM, Logistic Regression	Transformer-based embeddings, linguistic features	custom dataset of 12K Arabic tweets	BERT outperformed benchmark systems
[7] 2022	XGBoost, SVM, Genetic Algorithm (GA) optimization	Fine-tuned word embeddings	Arabic Cyberbullying Corpus (ArCybC) [45]	Hybrid approach achieved optimal performance
[8] 2023	Single and ensemble machine learning classifiers	Linguistic and statistical features	L-HSAB [25], ArCybC [45], and a custom Facebook comment dataset	Ensemble methods outperformed single learners
[9] 2021	BiLSTM, CNN, FastText, SVM, and NaiveBayes (NB)	Multilingual and dialectical features	Algerian Arabic Facebook of 8.7K comments	BiLSTM demonstrated the highest performance
[10] 2021	multilingual and Arabic BERT	original texts	Aljazeera dataset [24], YouTube dataset [22], L-HSAB [25], and OSACT4 [44]	Arabic monolingual BERT models outperform multilingual BERT
[11] 2022	BiLSTM	Domain-specific and domain-agnostic embeddings	custom dataset of over 500K Arabic tweets	BiLSTM achieved the best performance
[46] 2022	BERT-based transfer learning	Dialect-specific linguistic features	OSACT4 [44], ArCov19 [47], AJGT [48], and Hate Speech and Offensive Content Dataset [16]	BERT improves performance on the Tunisian and the Egyptian Arabic dialects
[12] 2021	transfer learning	syntactic and semantic features	custom dataset of 9K Arabic tweets	transfer learning improved Hebrew classification
