# Supplementary material for: Unveiling the spectrum of Arabic offensive language: Taxonomy and insights
Source: PLoS One. 2025 Jun 17;20(6):e0319900. doi: 10.1371/journal.pone.0319900 (PMC12173391; doi:10.1371/journal.pone.0319900)
Supplement: Attachment — Submitted filename: Response-letter.docx.pdf [file pone.0319900.s002.pdf]

Response to reviewers

We are very grateful to the reviewers and the academic editor for their comments and suggestions on our manuscript, PONE-D-24-33044, titled “Unveiling the Spectrum of Arabic Offensive Language: Taxonomy and Insights,” which have allowed us to improve the quality of our work. We describe below how we addressed all the issues reported by the editor and reviewers. All the changes are marked in the updated manuscript in blue font color.

Editorial comments

COMMENT 1: Please ensure that your manuscript meets PLOS ONE's style requirements, including those for file naming. The PLOS ONE style templates can be found at https://journals.plos.org/plosone/s/file?id=wjVg/PLOSOne_formatting_sample_main_body.pdf and https://journals.plos.org/plosone/s/file?id=ba62/PLOSOne_formatting_sample_title_authors_affiliations.pdf

RESPONSE 1: We have ensured that we use PLOS ONE latex template to format our manuscript.

COMMENT 2: In your Methods section, please include additional information about your dataset and ensure that you have included a statement specifying whether the collection and analysis method complied with the terms and conditions for the source of the data.

RESPONSE 2: We have added the following statement to the beginning of Section 4 that describes the data:

All datasets analyzed in this study were collected from publicly available sources and used in accordance with their respective terms and conditions. The collection and analysis of the data comply with ethical research standards and are restricted to publicly accessible content intended for research purposes.

COMMENT 3: Your abstract cannot contain citations. Please only include citations in the body text of the manuscript, and ensure that they remain in ascending numerical order on first mention.

RESPONSE 3: We have removed citations from the abstract and checked the manuscript to ensure that citations remain in ascending numerical order.

COMMENT 4: The overall research work is good and the authors are required to address the reviewers' comments. The authors have added table for comparison of datasets and the evaluation of results. A table of comparison of literature review is also expected sharing the details, such as Ref no, Year of Research work to show the chronological order of the work, methods used, features used for analysis, name of dataset and results. Thank you

RESPONSE 4: Following the suggestion, we added the table summarizing the works on offensive language detection in Arabic, covered in Section 2.1, to the Appendix and referenced it at the end of the section. The table was placed in the Appendix to prevent its size from interrupting the flow of the related work discussion.

ADDITIONAL MODIFICATIONS:

We have ensured that dataset sizes reported in the paper are consistent.

Reviewer #1

RECOMMENDATION #1: Expand on Implicit Language: Future research should aim to explore implicit offensive language more thoroughly. As this is a significant area in offensive communication, addressing it would provide a more complete understanding of the phenomenon.

RESPONSE #1: This is indeed one of our future research directions. The generalized offensive language taxonomy will be extended to cover implicit language. This task presents greater challenges than explicit language detection, as implicit offensive language is more culturally dependent and significantly harder to identify. Additionally, the lack of dedicated Arabic datasets for implicit offensive language presents a further obstacle. To address this issue, we have added the following paragraph to the Conclusions and Limitations section:

> *The future work will also focus on detecting implicit offensive language in Arabic, which poses a bigger challenge due to the subtlety and context-dependency of such expressions. Moreover, the lack of implicit offensive language datasets for Arabic poses an additional challenge.*

RECOMMENDATION #2: Dataset Unification: Consider developing a unified, annotated dataset that covers the wide spectrum of Arabic dialects, as this would improve the generalizability of model performance.

RESPONSE #2: Indeed, in this paper, we focused on MSA and the Levantine dialect. Other dialects that appear in publicly available datasets include Egyptian

(including Gulf), Maghrebi, Darija, and Saudi, but these do not cover the full variety of Arabic dialects. The main issue with these datasets is that some contain a mixture of dialects without any dialect annotation. However, this data can be used to improve the robustness of models for offensive language detection. We have added a statement to the Conclusions section outlining this as one of our future research goals:

> *As part of our future work, we aim to explore the use of mixed-dialect datasets, despite their lack of dialect annotations, to enhance the robustness of models for offensive language detection across a broader range of Arabic dialects.*

RECOMMENDATION #3: Fine-Tuning Insights: Offering more detailed recommendations on fine-tuning models for specific Arabic dialects would improve practical applications for offensive language detection systems.

RESPONSE #3: Following your suggestion we added these recommendations to the end of Section 5.2.2 that describes fine-tuned models performance:

> *Based on the results of fine-tuning for Arabic offensive language detection presented in Table 20, we suggest the following recommendations for enhancing model performance when targeting specific Arabic dialects.:*
>
> > *• To incorporate preprocessing techniques tailored to specific dialects, such as normalizing dialectal variations and unique morphological patterns.*
> >
> > *• To augment datasets with synthetic examples that reflect diverse dialectal features can help mitigate data scarcity issues.*
> >
> > *• To apply dialect-specific transfer learning by fine-tuning models on datasets that are exclusively representative of a target dialect before applying them to mixed or broader datasets could lead to more effective feature learning.*

Reviewer #2

RECOMMENDATION #1: Applications and Advantages: The authors should include a paragraph in the introduction highlighting the practical applications and benefits of the proposed work. This will help contextualize its significance.

RESPONSE #1: Thank you for your suggestion. We added the following paragraph to the Introduction:

There are several real-world uses for offensive language detection in the Arabic language, such as content moderation, online community management, and social media surveillance. We strive to improve the accuracy of automated offensive detection systems by creating a uniform taxonomy and joining multiple existing datasets. Furthermore, considering the wide linguistic variety of the Arabic language in different countries, our work can facilitate the creation of dialect-specific detection models, which are currently underdeveloped.

RECOMMENDATION #2: Improved Contributions Section: The contribution paragraph should be revised to explicitly specify the techniques or added features that contribute to the improved accuracy of the proposed models.

RESPONSE #2: We rewrote the contributions part of the Introduction to make more emphasis on the advantages of our approach. This is the new contributions section:

This paper introduces a novel offensive language taxonomy tailored for Arabic, which enhances the granularity and relevance of dataset annotations (Section 3). We provide a detailed description of each level of the taxonomy, highlighting its significance in improving dataset analysis and facilitating cross-dialectal studies (Section 4). Our analysis covers 17 datasets, including re-annotated and filtered versions, and introduces a new combined dataset for Modern Standard Arabic (MSA) and Levantine dialects (Section 5.3.3).

To improve model accuracy, we evaluate pre-trained and fine-tuned Arabic transformer models, applying techniques such as transfer learning across dialects and fine-tuning on re-annotated datasets to capture nuanced patterns in offensive language (Section 5.2). Our experiments demonstrate the efficacy of combining dialect-specific datasets with the proposed taxonomy in enhancing classification performance. Finally, we discuss the broader implications of our findings for advancing research in Arabic offensive language detection and outline potential directions for future work (Section 6).

RECOMMENDATION #3: Enhanced Related Work Section: The related work section can be strengthened by connecting studies based on publication year or thematic similarities. Rather than simply referencing studies as "the paper [23]" or "the study in [42]," the authors could cite them using author names (e.g., "Levi et al.") or mention the models or approaches discussed in those studies.

RESPONSE #3: We have revised our related work section by organizing the studies based on publication year, as suggested. Also, we now cite authors explicitly, as per your recommendation.

RECOMMENDATION #4: Pictorial Representation: Including a pictorial representation of the study's steps or framework would significantly improve readers' understanding of the proposed methodology.

RESPONSE #4: Following your suggestion, we added a new Section 5.1 titled "Pipeline" to the paper, which includes a description and a figure illustrating our methodology:

> *The primary stages of our approach are illustrated in Figure 1. We begin by using re-annotated and filtered datasets, as detailed in Section 4.1. Next, we analyze each dataset individually, along with a combined MSA and Levantine dataset, which we construct as described in Section 5.3.3. We assess the performance of each model (outlined in Section 5.2) with and without fine-tuning on these datasets and present the results.*

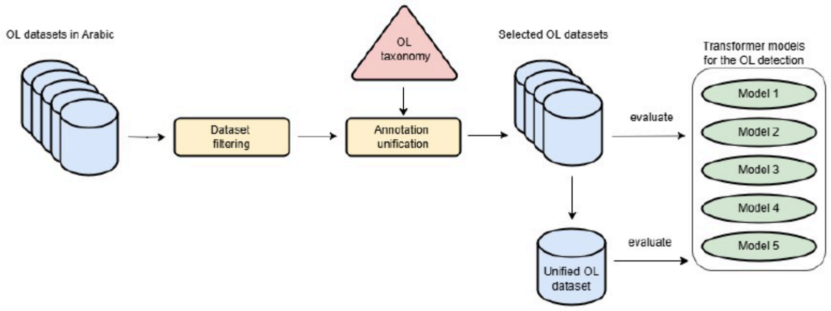

Fig 1. Evaluation pipeline

RECOMMENDATION #5: Results Presentation: The results section can be improved by incorporating a mix of graphical and tabular representations. Graphs can provide a visual comparison, while tables, as seen in Tables 9-19, can offer detailed numerical insights, facilitating a comprehensive performance analysis against baseline models.

RESPONSE #5: To enhance the presentation of results, we added two charts to Sections 5.3.1 and 5.3.2. These charts compare the performance of all tested models (with and without fine tuning) across all datasets, illustrating how model accuracy varies between datasets and how the models perform relative to each other. We show the charts below:

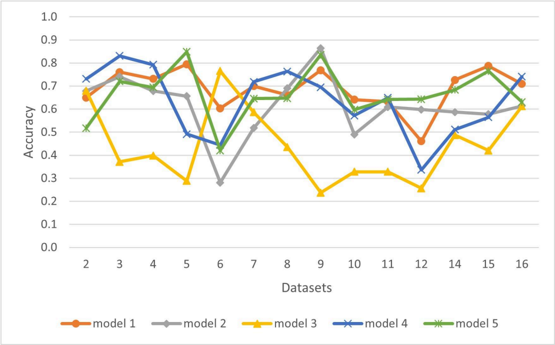

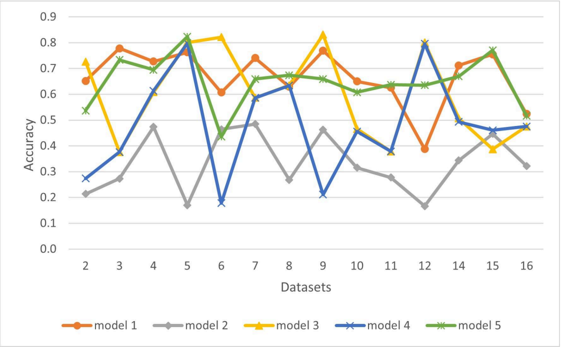

RECOMMENDATION #6: Future Work: Authors can improve the future work with more concrete research directions.

RESPONSE #6: We added two concrete research directions to our Conclusions section:

1. *As part of our future work, we aim to explore the use of mixed-dialect datasets, despite their lack of dialect annotations, to enhance the robustness of models for offensive language detection across a broader range of Arabic dialects.*

2. *Future research can explore the adaptation of the proposed taxonomy or its core principles for offensive language detection in other languages, fostering a more inclusive online environment globally. It will also focus on detecting implicit offensive language in Arabic, which poses a bigger challenge due to the subtlety and context-dependency of such expressions. Moreover, the lack of implicit offensive language datasets for Arabic poses an additional challenge.*
